# Photon-Counting Detector CT Virtual Monoenergetic Images in Cervical Trauma Imaging—Optimization of Dental Metal Artifacts and Image Quality

**DOI:** 10.3390/diagnostics14060626

**Published:** 2024-03-15

**Authors:** Daniel Dillinger, Daniel Overhoff, Matthias F. Froelich, Hanns L. Kaatsch, Christian Booz, Achim Hagen, Thomas J. Vogl, Stefan O. Schönberg, Stephan Waldeck

**Affiliations:** 1 Department of Vascular Surgery and Endovascular Surgery, Bundeswehr Central Hospital, Rübenacher Straße 170, 56072 Koblenz, Germany; 2 Department of Radiology and Neuroradiology, Bundeswehr Central Hospital, Rübenacher Straße 170, 56072 Koblenz, Germany; 3 Department of Radiology and Nuclear Medicine, University Medical Centre Mannheim, Medical Faculty Mannheim, Heidelberg University, Theodor-Kutzer-Ufer 1-3, 68167 Mannheim, Germany; 4 Institute for Diagnostic and Interventional Radiology, Goethe-University, Theodor-Stern-Kai 7, 60590 Frankfurt am Main, Germany; 5 Department of Neuroradiology, University Medical Center Mainz, Langenbeckstraße 1, 55131 Mainz, Germany

**Keywords:** computed tomography, metal artifacts, virtual monoenergetic imaging, spectral imaging, photon counting detector CT

## Abstract

Objectives: The aim of this study was to analyze the extent of dental metal artifacts in virtual monoenergetic (VME) images, as they often compromise image quality by obscuring soft tissue affecting vascular attenuation reducing sensitivity in the detection of dissections. Methods: Neck photon-counting CT datasets of 50 patients undergoing contrast-enhanced trauma CT were analyzed. Hyperattenuation and hypoattenuation artifacts, muscle with and without artifacts and vessels with and without artifacts were measured at energy levels from 40 keV to 190 keV. The corrected artifact burden, corrected image noise and artifact index were calculated. We also assessed subjective image quality on a Likert-scale. Results: Our study showed a lower artifact burden and less noise in artifact-affected areas above the energy levels of 70 keV for hyperattenuation artifacts (conventional polychromatic CT images 1123 ± 625 HU vs. 70 keV VME 1089 ± 733 HU, *p* = 0.125) and above of 80 keV for hypoattenuation artifacts (conventional CT images −1166 ± 779 HU vs. 80 keV VME −1170 ± 851 HU, *p* = 0.927). Vascular structures were less hampered by metal artifacts than muscles (e.g., corrected artifact burden at 40 keV muscle 158 ± 125 HU vs. vessels −63 ± 158 HU *p* < 0.001), which was also reflected in the subjective image assessment, which showed better ratings at higher keV values and overall better ratings for vascular structures than for the overall artifact burden. Conclusions: Our research suggests 70 keV might be the best compromise for reducing metal artifacts affecting vascular structures and preventing vascular contrast if solely using VME reconstructions. VME imaging shows only significant effects on the general artifact burden. Vascular structures generally experience fewer metal artifacts than soft tissue due to their greater distance from the teeth, which are a common source of such artifacts.

## 1. Introduction

**Figure 1:**
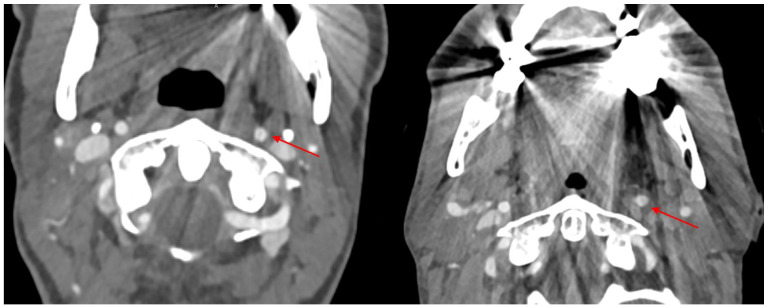
Two different patients. In both, metal artifacts hamper the image quality and mock a dissection in the left inner carotid artery (marked with red arrows).

Patients in the emergency room after severe trauma are often assessed with CT scans to check for trauma-related injuries [1]. CT imaging is also an important diagnostic modality for inflammation and tumors. Dental artifacts often interfere with the image quality by causing hyperdense and hypodense artifacts (caused by beam hardening artifacts from absorbing low-energy photons and photon starvation from complete photon absorption) and increased image noise (scatter artifacts from large attenuation differences in metal and surrounding soft tissue) [2,3,4,5,6,7]. The extent of metal artifacts depends on the image acquisition and reconstruction parameters as well as the material itself. Artifacts can lead to reduced diagnostic quality or even mimic pathological conditions. (e.g., dissections, which can be seen in Figure 1). In order to overcome this, various techniques in conventional CT have been introduced. Metal artifacts can be reduced by modulating either the tube voltage and/or current as well as changes in the collimation. This can lead to increased radiation dose concomitant with greater risks to the patients [8]. Dual-energy CT technologies have shown effects on metal artifact reduction in past research using VME imaging [9,10]; in addition, tin prefiltration has also been used in previous studies for artifact reduction [11,12]. Along with the acquisition of spectral CT datasets from the latest generations of CT devices, VME images can be reconstructed and used for metal artifact reduction. In measuring the deposited energy of the arriving photon directly on the photon counting detector (PCD) CT, each scan has this inherent spectral information and shows less electronic noise than previous detector technologies and can offer a higher spatial resolution [13,14,15,16]. All these advantages of PCD-CT can lead to increased image quality, less artifacts and noise as previous research has shown [13,14,16,17]. The aim of this study was to evaluate the effects of different VME thresholds on metal artifacts caused by dental hardware in trauma CT scans, regarding the assessability of vessels and muscle tissue.

## 2. Materials and Methods

A local ethics committee (Mainz, Germany) approved this study, which was conducted in accordance with the Declaration of Helsinki. Written consent was waived due to the retrospective design of the study; no scan was performed only for this research.

### 2.1. Study Population

We included 50 patients aged 18 or more, undergoing a clinically indicated CT scan for trauma on a first generation PCD-CT (NAEOTOM Alpha Siemens Healthineers, Forchheim, Germany) with relevant dental metal artifacts (with severe affection of the adjacent soft tissue) between April 2022 and August 2022. Patients with motion artifacts or missing spectral datasets were excluded.

### 2.2. Imaging Protocol

All patients were scanned after acquiring two topograms and by modulating the tube current in the z-axis in accordance with the patient’s diameter and attenuation, which was calculated using the topograms.

Patients received contrast medium (Xenetix 350, containing 76, 78 g/100 mL Iobitridol) through an antecubital vein with a power injector (CT Motion XD8000, Ulrich Medical, Ulm, Germany). In trauma, we use a double bolus technique of 60 mL contrast medium (flow 4 mL/s) followed by a saline chaser of 50 mL (flow 4 mL/s); after a pause of 15 s, the same injection is repeated. The head and neck scan was started with bolus tracking in the thoracic descending aorta and a predefined threshold of 100 HU (using CARE Bolus software (version VA50A), Siemens Healthineers, Forchheim, Germany) with a delay of 15 s. The scans were performed with CARE keV, image quality level: 145.

The data were acquired at 120 keV, and our scans were performed in a the cranio–caudal direction with a pitch of 0.80 and an increment of 0.70 mm.

Spectral post-processing (SPP) datasets were analyzed with a slice thickness of 1 mm in a Qr40 kernel.

We also retrieved the dose-length product (DLP) and CT dose index (CTDIvol), and as a replacement for BMI, we calculated the effective patient diameter (Deff) according to O’Neil et al. [18].

### 2.3. Objective Image Analysis

Two independent readers (with ten and twelve years of experience in trauma CT) analyzed the datasets with dedicated software (Syngio.via, version VB60A_HF04, Siemens Healthineers, Forchheim, Germany) in a Monoenergetic+ application profile. Regions of interest (ROIs) were positioned by each reader in most hyper- and hypodense artifacts radiating from dental metal implants, from muscle and arteries affected by these artifacts and in normal muscle tissue and arteries by each reader. ROIs were placed in the same area first using VME imaging and then copied to polychromatic images to ensure that they were in comparable positions. In the VME images, the ROIs remained consistent across all energy levels due to automated processing. The ROIs had sizes of at least 0.5 cm^2^. Both readers were given the freedom to select the CT slice that they believed exhibited the most significant hyper- and hypodense artifacts (Supplementary Figure S1). The attenuation (in Hounsfield units (HUs)) and standard deviation (SD) were acquired. The SD was considered image noise in the corresponding measurement. The VME images were reconstructed on the energy levels of 40–190 keV in 10 keV increments, and the HUs and SD were acquired. Besides that, we also used polychromatic conventional images (CIs) for the corresponding measurements and placed ROIs in the same area. We also placed a ROI in non-affected subcutaneous fat, to calculate the corrected image noise (CIN) (correction for less image noise at higher energy levels), as previously reported by Große Hokamp et al. (with CIN = SD − SDfat) [19]. We also calculated the difference of attenuation in corresponding artifacts in affected and non-affected areas as the corrected artifact burden (∆HU = HUartifact − HUno artifact), as well as the artifact index (AIX) (√(SD2artifact − SD2no artifact)), as described in previous studies [20,21,22].

### 2.4. Subjective Image Analysis

**Figure 2:**
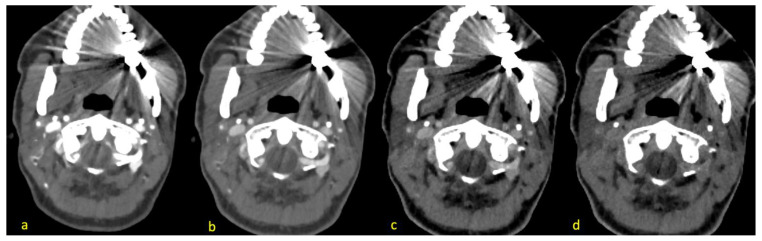
Different reconstructions of the energy levels of 40 keV (**a**), 70 keV (**b**), 110 keV (**c**) and 190 keV (**d**) of the same patient with hyperattenuation artifacts radiating from dental hardware and hypoattenuation artifacts facing mostly mediodorsal and anterolateral (opposing each other). Vascular contrast decreases with higher energy levels.

The same readers who conducted the objective analysis rated the subjective image quality on the energy levels of 40, 70, 110 and 190 keV on two Likert scales, which are demonstrated in Table 1. The increments were picked as described before to make sure that differences in the images could be discovered by avoiding subtle changes caused by too-close energy thresholds [23]. Also, the readers checked for new appearing artifacts from the VME reconstructions. Figure 2 shows the effects of the different reconstructions. The readers were free to change the window settings.

### 2.5. Statistical Analysis

Statistics were calculated with SPSS (version 27, IBM Corporation, Armonk, NY, USA). We evaluated ordinals as absolute numbers including their percentages. Interval variables were reported as means and standard deviations. An analysis of variance (ANOVA) a post hoc *t*-test for paired samples, was performed. A *p*-value less than 0.05 was considered statistically significant. Inter-reader agreement was calculated using Cohen’s Kappa. We considered a Kappa of 0 as poor agreement, between 0.00 and 0.20 as slight agreement, from 0.21 to 0.4 as fair agreement, 0.41–0.6 as moderate agreement, 0.61–0.8 as substantial agreement and 0.81–1.00 as almost perfect, according to Landis et al. [24].

## 3. Results

### 3.1. Population Data

We analyzed the datasets of 50 patients, of which 32 (64%) were male and 18 (36%) were female. The epidemiologic data are presented in Table 2.

### 3.2. Hyperattenuation Artifacts

**Figure 3:**
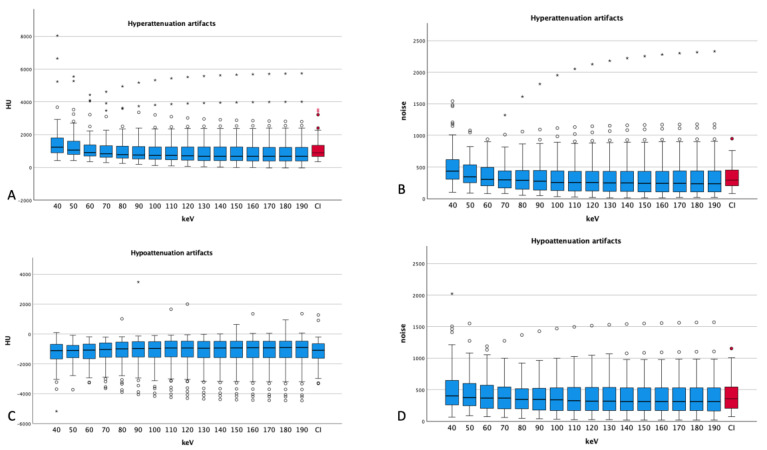
Attenuation and noise of hyper- and hypoattenuation artifacts ((**A**) attenuation, (**B**) noise for hyperattenuation artifacts; (**C**) attenuation and (**D**) noise for hypoattenuation artifacts). Dots and asterisks show aberrant values. For both types of artifacts, one aberrant measurement can be seen with increasing noises for higher energy levels (CI: conventional images, in red); the ANOVA for hyperattenuation artifacts was *p* < 0.001; for hypoattenuation artifacts, *p* = 1.000; for noise in hyperattenuation artifacts, *p* = 0.002; and in hypoattenuation artifacts, *p* = 0.246.

Hyperattenuation artifacts showed a decreasing mean attenuation with increasing energy levels (from 40 keV 1520 ± 1130 HU to 90 keV 904 ± 856 HU). Further details can be seen in Figure 3. A few measurements showed increasing HU values in hyperattenuation artifacts with increasing keV, which is also pointed out in Figure 3.

The mean noise in hyperattenuation artifacts showed a maximum at 40 keV (506 ± 294 HU) and decreased with higher energy levels to 330 at 120 keV and above. Compared with the conventional images, hyperattenuation artifacts showed lower values for the energy levels of 70 keV and above (CI 1123 ± 625 HU vs. 70 keV 1089 ± 733 HU, *p* = 0.125) and noise showed lower values for the levels of 80 keV and above (CI 343 ± 184 HU vs. 80 keV 338 ± 255 HU, *p* = 0.703), but 70 keV (346 ± 228 HU) shows only slightly worse values than CI with no statistical significance (*p* = 0.865). The difference in attenuation comparing VME images with CIs reaches statistical significance below the energy levels of 50 keV (*p* < 0.001) and above 80 keV (*p* = 0.005 and lower). Noise shows only significant differences for the VME levels of 40 and 50 keV compared to that for CIs (*p* < 0.001).

### 3.3. Hypoattenuation Artifacts

Hypoattenuation artifacts showed only a slight increase in mean attenuation with increasing keV (from 40 keV −1257 ± 875 HU to 190 keV −1135 ± 1009 HU). Noise in hypoattenuation artifacts peaks at 40 keV (497 ± 337 HU) and reaches a plateau at 100 keV and above (at around 384 HU). Figure 3 also demonstrates the attenuation and noise of hypoattenuation artifacts.

In comparing CIs to VME images, CIs show an average of −1166 ± 779 HU for hypoattenuation artifacts; the energy levels of 80 keV and above show comparable values (CI −1166 ± 779 HU vs. 80 keV −1170 ± 851 HU, *p* = 0.927).

Noise shows lower values than CI for 80 keV and above (CI 404 ± 239 HU vs. 80 keV 391 ± 253 HU, *p* = 0.218), but also here, 70 keV (CI 404 ± 239 HU vs. 405 ± 253 HU, *p* = 0.851) shows no statistically significant difference compared to that in CIs. No significant differences could be found comparing CIs with VME reconstructions regarding attenuation, but noise showed significantly worse values at 40 keV (497 ± 337 HU, *p* < 0.001) and 50 keV (436 ± 269 HU, *p* = 0.037) than in CIs. The other *t*-tests showed no significant differences regarding noise.

### 3.4. Artifacts and Noise in Muscle

**Figure 4:**
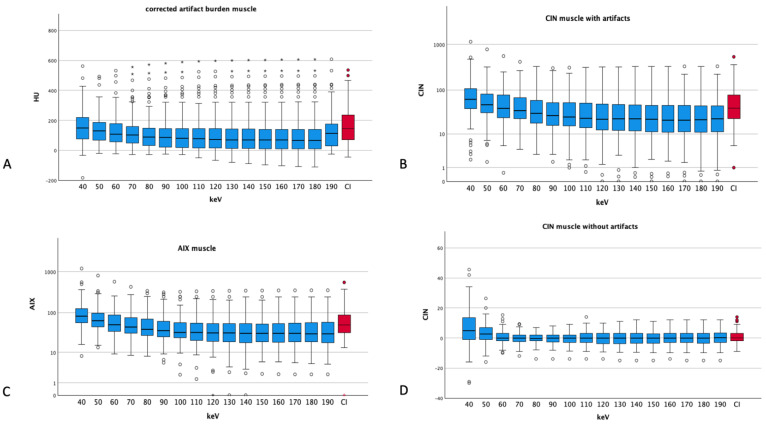
Different artifact indices of muscle tissue ((**A**) corrected artifact burden, (**B**) CIN muscle with artifacts, (**C**) AIX muscle and (**D**) CIN of muscles without artifacts). Dots and asterisks represent aberrant and even more aberrant values. (**B**,**C**) show a logarithmic scale for a better visualization (CI: conventional images, in red, HU: Hounsfield units, CIN: corrected image noise, AIX: artifact index). The ANOVA conducted for the corrected artifact burden showed a *p* of 0.077; the rest showed *p* values smaller than 0.001.

The corrected artifact burden for muscle showed the highest value for 40 keV for all VME images (158 ± 125 HU) and the lowest at the energy thresholds of 180 keV (104 ± 137 HU) with increasing values at 190 keV again (132 ± 127 HU), but CIs showed an even higher artifact burden than all VME reconstructions (160 ± 126 HU) (see Figure 4).

The CIN for normal muscle undulates around 0 with a maximum at 40 keV (5.44 ± 12.83 HU) and a minimum at 110 keV (0.03 ± 4.64 HU). In comparison with Cis, which show 0.43 ± 4.37 HU, the CIN absolute values of the energy levels of 70 keV to 180 keV are lower than those of CIs.

The AIX showed the highest values for muscle at 40 keV (117.92 ± 139.89) with a plateau at around 50 for energy thresholds of 110 keV and above. CIs showed an AIX of 70.87 ± 72.51, which is worse than the AIX of the VME reconstructions of 60 keV (69.82 ± 69.53) and above.

### 3.5. Artifacts and Noise in Vessels

The corrected vascular artifact burden showed the highest absolute values at 40 keV (−63 ± 158 HU), reaching 0 ± 50 HU at the energy level of 130 keV, and from there, a slight increase up to 6 ± 57 HU at 180 keV can be seen. Compared to CIs, the artifact burden showed lower values starting from the energy levels of 60 keV (CI − 23 ± 85 HU vs. 60 keV −22 ± 91 HU).

For vessels, the CIN showed a steady decrease with higher energy levels, starting from 40 keV (97 ± 68 HU) and reaching a plateau at 150 keV and above (4 to 5). CIs showed an average of 40.90 ± 28.00, which exceeded the VME reconstructions of 70 keV (28.53 ± 20.85 HU) and above.

For vessels, the AIX also showed decreasing values starting from 40 keV (121.55 ± 94.70), reaching a stable level at around 20–22 at 110 keV and above. In comparison with CIs (49.96 ± 30.42), the VME reconstructions had lower values at the energy levels of 60 keV (50.33 ± 31.46) and above.

### 3.6. Comparison of Muscle and Vessels

**Figure 5:**
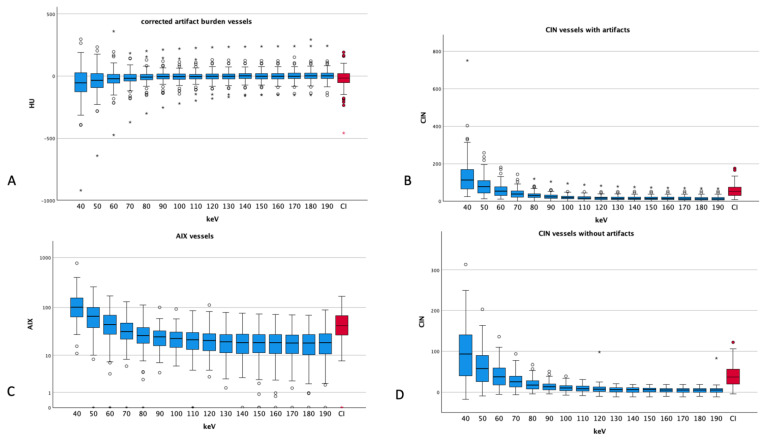
Different artifact indices of vessels ((**A**) corrected artifact burden, (**B**) CIN vessels with artifacts, (**C**) AIX muscle and (**D**) CIN of vessels without artifacts). Dots and asterisks represent aberrant and even more aberrant values. (**B**) shows a logarithmic scale for a better visualization (CI: conventional images, in red, HU: Hounsfield units, CIN: corrected image noise, AIX: artifact index). All ANOVAs showed *p*-values less than 0.001.

Vessels showed a lower corrected artifact burden compared to muscle at all energy thresholds (e.g., 40 keV muscle 158 ± 125 HU vs. vessels −63 ± 158 HU *p* < 0.001 on all tested energy levels) (see Figure 5). Regarding the AIX, vessels showed overall lower values besides at the level of 40 keV (vessels 121.55 ± 94.70 vs. muscle 117.92 ± 139.89, *p* = 0.795), reaching statistical significance above the level of 60 keV (*p* = 0.015 and less). The CIN for vessels showed higher values at the energy thresholds of 40 and 50 keV compared to muscle tissue (*p* = 0.024 for 40 keV, *p* = 0.227 for 50 keV), reaching almost equal values at 60 keV (muscle 59.17 ± 68.81 HU vs. vessels 57.55 ± 34.68 HU, *p* = 0.845). Above 60 keV, vessels showed less CIN than muscle tissue, reaching statistically significant levels at 80 keV (*p* = 0.005 and less for the higher keV levels).

### 3.7. Subjective Analysis

**Figure 6:**
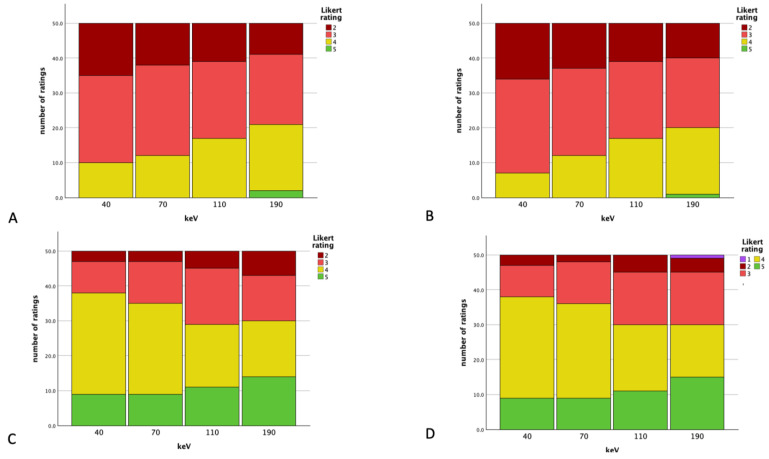
Rating results for the overall artifact burden for the different readers (**A**,**B**) and vessel artifact burden for the different readers (**C**,**D**) at the different energy thresholds.

A subjective analysis showed a Cohen’s Kappa of 0.782 regarding the overall artifact burden (substantial inter-reader agreement), the vascular assessability showed an almost perfect inter-reader agreement (Cohen’s Kappa: 0.817). Figure 6 visualizes the ratings at the different energy thresholds. Higher keV levels showed better subjective ratings; vascular structures showed higher ratings than the overall artifact burden. In summing up all the Likert ratings for the overall artifact burden at 40 keV of reader one, a total of 145 could be calculated; reader two had 141 (overall: 286) for vascular artifacts, and a total of 388 was seen (both readers: 194). The energy level of 70 keV showed a total of 299 for the overall artifacts by summing up all ratings (reader one: 150, reader two: 149) and 384 for vascular artifact affection (reader one: 191, reader two: 193). At the energy level of 110 keV, the subjective overall artifact burden scored a total of 312 (reader one: 156 and reader two: 156 as well), and the vascular artifact burden, a total of 371 (reader one: 185, reader two: 186). The level of 190 keV showed 164 for reader one and 161 for reader two (total of 325), and regarding the overall artifact burden, 187 for reader one and 189 for reader two (total of 376) for vascular artifact affection.

None of the readers discovered new artifacts caused by the VME reconstruction on the examined energy levels.

## 4. Discussion

The aim of this study was to evaluate the impact of VME reconstructions on hyperdense and hypodense artifacts caused by dental hardware with a special focus on muscles and neck vessels.

Our research showed a lower noise in artifact-burdened areas compared to conventional images as well as a lower general artifact burden starting from the VME levels of 70/80 keV. Lower energy thresholds showed more artifacts, and higher energy levels showed fewer metal artifacts, which is concordant with the subjective image analysis. The subjective analysis showed increasing values for the overall artifact burden, whereas vascular artifact affection was rather stable at all energy levels.

Overall, vessels showed a lower artifact burden than the adjacent muscles, which can be explained by the distance of the carotid arteries to the dental hardware, therefore resulting in less artifacts, which is also represented in the above-mentioned subjective image analysis for the vascular artifact burden (higher scores for vascular artifact affection compared to the overall artifact burden and only minor changes in the overall scores for vascular artifacts were observed).

Due to contrast medium effects, artifact-affected vessels show higher absolute values for CIN compared to muscle at lower energy levels. Previous studies have also shown worse vascular assessability with increasing energy thresholds (because of decreasing vascular attenuation) [25], but the present study already showed a lower vascular artifact burden at the energy level of 60 keV and above. This decreasing attenuation with higher energy levels as well as monoenergetic+ mode [26] could be a reason for the CIN undulating around 0 at higher energy thresholds for both artifact-affected and non-affected vascular structures. The previously described best vascular contrast at 70 keV can be used to decrease artifact effects on vascular structures. Further reconstructions at higher energy levels result in less vascular contrast and also less artifact reductions, as previously described, and therefore leading to no relevant benefit [26].

Risch et al. [21] as well as Patzer et al. [27] showed greater effects of metal reduction for specialized metal reduction reconstruction algorithms like IMAR compared to VME reconstructions. Risch et al. showed only statistic significant changes if the reconstruction was performed with metal artifact reconstruction algorithms, which is in line with our research. However, neither took contrast medium effects and visualization of vessels into account. Our research showed better values for attenuation and noise in hyper- and hypoattenuation artifacts at the energy levels of 80 keV and above (even at 70 keV in hyperattenuation artifacts regarding attenuation); the break-even point is also described in the research of Patzer et al. [27].

The loss of contrast medium effects is the reason why the results of this study differ from those of similar research, which found 130 keV to have the most effects of VME imaging on the extent of metal artifacts caused by dental hardware [28].

Tin prefiltration might lead to another benefit regarding metal artifact reduction as previous studies described; further studies will have to evaluate the effects of spectral shaping especially on vascular structures and on PCD-CT, as previous studies have examined only non-contrast-enhanced scans [12] or datasets from dual-source CT scanners [11].

Our study has certain limitations. First of all, we did not use metal artifact reduction reconstructions, which could be used to further reduce the effects of metal implants as previous studies have shown [21,27]; these studies showed the major effects of these algorithms compared to those of VME reconstruction. Also, we did not differentiate between heavy- and low-dental material artifacts in our cohort, which could especially affect the subjective ratings, as well as the effects of the VME reconstructions, as Zhu et al. described for spectral cone-beam CT [22].

Additionally, we did not evaluate the effects of different metal compositions on the artifacts, as different materials might also influence the extents of the resulting metal artifacts.

Another limitation is that we used only one scanning protocol with one contrast medium injection protocol. This adds to the comparability of the images, but the examination of metal artifacts from more scanning protocols could add valuable information. Furthermore, the results are also limited because this is a single-center retrospective study.

The present study shows only the minor effects of virtual monoenergetic reconstructions on metal artifacts caused by dental implants and fillings in polytrauma scans, higher energy levels showing less artifacts but also less vascular contrast and why the levels of 70 and 80 keV seem to be the best tradeoff if using only VME reconstruction for artifact reduction. The artifacts mostly affect the surrounding soft tissue; vascular structures are less hampered by these metal artifacts due to their distance to the artifact-causing dental hardware.

## Figures and Tables

**Table 1:** Likert ratings of the overall and vascular artifact burden and the consecutive subjective image quality; ratings 3–5 provide sufficient diagnostic quality.

Rating	Overall Image Quality/Artifact Burden	Vascular Assessability/Vascular Artifact Burden
1	massive artifacts; very poor image quality/no diagnostic use	no diagnostic assessability of neck vessels
2	pronounced streaks; poor image quality/very limited diagnostic assessability	Severe impairment of the diagnostic assessability
3	intermediate streaks; intermediate image quality/restricted diagnostic assessability	Moderate impairment, but sufficient diagnostic assessability
4	minimal streaks; good image quality/sufficient diagnostic assessability	minor impairment
5	no artifacts; excellent image quality/diagnostic assessability	excellent assessability

**Table 2:** Ages, radiation data and effective patient diameters (Deff) of the study population (*n* = 50) including differentiation by sex.

	Overall	Male	Female
Age (years)	63 ± 16	59 ± 14	70 ± 18
DLP (mGy·cm)	318 ± 40	334 ± 28	291 ± 43
CTDI_vol_ (mGy)	7.77 ± 0.74	8.00 ± 0.50	7.34 ± 0.89
D_eff_ (mm)	306 ± 53	311 ± 42	297 ± 69

## Data Availability

The data of the current research can be requested from the corresponding author.

## Supplementary Materials

The following supporting information can be downloaded at https://www.mdpi.com/article/10.3390/diagnostics14060626/s1, Figure S1 shows the placement of the different ROIs (ROI 1: muscle artifacts, ROI 2: hypoattenuation artifact, ROI 3: hyperattenuation artifact); the ROIs of non-affected areas and vascular structures are not demonstrated on this slice; the readers were free to pick the slice that was the most appropriate.
