# Supplementary material for: Photon-Counting Detector CT Virtual Monoenergetic Images in Cervical Trauma Imaging—Optimization of Dental Metal Artifacts and Image Quality
Source: Diagnostics (Basel). 2024 Mar 15;14(6):626. doi: 10.3390/diagnostics14060626 (PMC10968735; doi:10.3390/diagnostics14060626)
Supplement: Supplementary file 1 [file diagnostics-14-00626-s001.zip › diagnostics-2861364-supplementary.pdf]

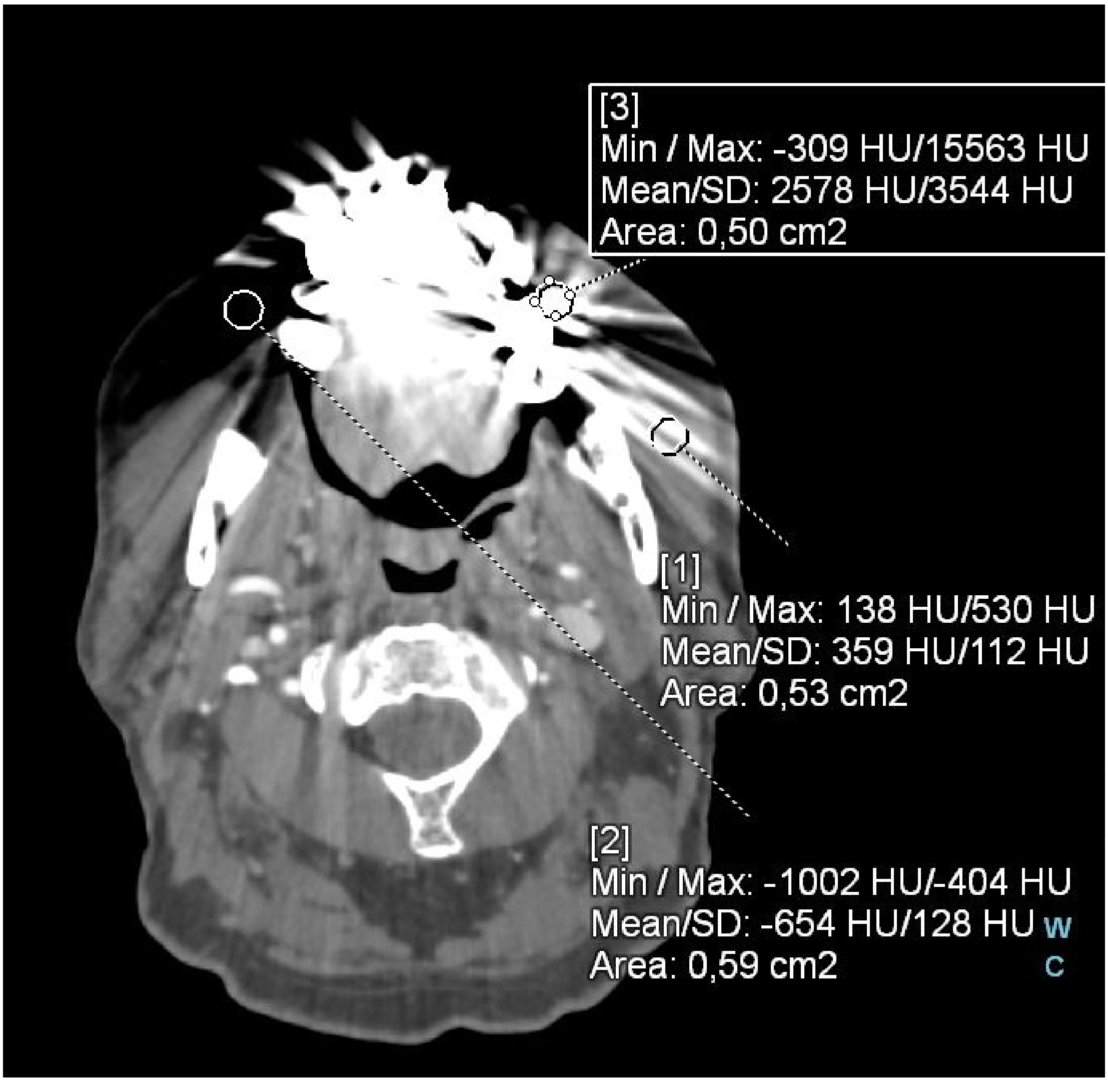


Supplemental Figure S1 shows the placement of the different ROIs (ROI 1: muscle artifacts, ROI 2: hypoattenuation artifact, ROI 3: hyperattenuation artifact); the ROIs of non-affected areas and vascular structures are not demonstrated on this slice; the readers were free to pick the slice that was the most appropriate.
